# Non-parametric Heat Map Representation of Flow Cytometry Data: Identifying Cellular Changes Associated With Genetic Immunodeficiency Disorders

**DOI:** 10.3389/fimmu.2019.02134

**Published:** 2019-09-11

**Authors:** Julia I. Ellyard, Robert Tunningley, Ayla May Lorenzo, Simon H. Jiang, Amelia Cook, Rochna Chand, Dipti Talaulikar, Ann-Maree Hatch, Anastasia Wilson, Carola G. Vinuesa, Matthew C. Cook, David A. Fulcher

**Affiliations:** ^1^Department of Immunology and Infectious Diseases, Australian National University, Canberra, ACT, Australia; ^2^Centre for Personalised Immunology, John Curtin School of Medical Research, Australian National University, Canberra, ACT, Australia; ^3^Department of Nephrology, The Canberra Hospital, Canberra, ACT, Australia; ^4^Department of Immunology, The Canberra Hospital, Canberra, ACT, Australia; ^5^Department of Hematology, The Canberra Hospital, Canberra, ACT, Australia

**Keywords:** flow cytometry, immunodeficiency, common variable immunodeficiency, TACI, CTLA4, TNFSF13B, CARD11

## Abstract

Genetic primary immunodeficiency diseases are increasingly recognized, with pathogenic mutations changing the composition of circulating leukocyte subsets measured by flow cytometry (FCM). Discerning changes in multiple subpopulations is challenging, and subtle trends might be missed if traditional reference ranges derived from a control population are applied. We developed an algorithm where centiles were allocated using non-parametric comparison to controls, generating multiparameter heat maps to simultaneously represent all leukocyte subpopulations for inspection of trends within a cohort or segregation with a putative genetic mutation. To illustrate this method, we analyzed patients with Primary Antibody Deficiency (PAD) and kindreds harboring mutations in *TNFRSF13B* (encoding TACI), *CTLA4*, and *CARD11*. In PAD, loss of switched memory B cells (B-SM) was readily demonstrated, but as a continuous, not dichotomous, variable. Expansion of CXCR5+/CD45RA- CD4+ T cells (X5-Th cells) was a prominent feature in PAD, particularly in TACI mutants, and patients with expansion in CD21-lo B cells or transitional B cells were readily apparent. We observed differences between unaffected and affected TACI mutants (increased B cells and CD8+ T-effector memory cells, loss of B-SM cells and non-classical monocytes), cellular signatures that distinguished *CTLA4* haploinsufficiency itself (expansion of plasmablasts, activated CD4+ T cells, regulatory T cells, and X5-Th cells) from its clinical expression (B-cell depletion), and those that were associated with *CARD11* gain-of-function mutation (decreased CD8+ T effector memory cells, B cells, CD21-lo B cells, B-SM cells, and NK cells). Co-efficients of variation exceeded 30% for 36/54 FCM parameters, but by comparing inter-assay variation with disease-related variation, we ranked each parameter in terms of laboratory precision vs. disease variability, identifying X5-Th cells (and derivatives), naïve, activated, and central memory CD8+ T cells, transitional B cells, memory and SM-B cells, plasmablasts, activated CD4 cells, and total T cells as the 10 most useful cellular parameters. Applying these to cluster analysis of our PAD cohort, we could detect subgroups with the potential to reflect underlying genotypes. Heat mapping of normalized FCM data reveals cellular trends missed by standard reference ranges, identifies changes associating with a phenotype or genotype, and could inform hypotheses regarding pathogenesis of genetic immunodeficiency.

## Introduction

Widespread availability of DNA sequencing has uncovered an expanding array of genetic explanations for primary immunodeficiency disorders (PIDs) (1). Causative mutations can change cellular function and affect the balance between protective immunity and immune tolerance, resulting in clinical phenotypes of recurrent infection with or without autoimmunity. Changes in cellular physiology frequently result in quantitative differences in the composition of circulating leukocyte populations, which can be determined using multiparameter flow cytometry (FCM); indeed the power of this technique to measure an increasing array of specific subpopulations has challenged the ability to depict and analyse potentially important and pathogenically relevant cellular changes that might accompany or even identify a disease phenotype or genotype.

Traditionally, quantitative variations in cell subpopulations have been deemed to be abnormal if they vary significantly from a background control population, with the target range calculated based on two standard deviations either side of the mean, or by using 95% confidence intervals if non-parametric in distribution. Whilst stringent, this approach has the potential to miss less extreme but physiologically relevant cellular changes when found consistently in a cohort of patients, defined either by clinical phenotype or genotype; such subtle cellular changes could be more readily identified if all relevant parameters could be visually presented simultaneously, allowing identification of trends that might be considered for subsequent statistical analysis, and analysis in larger cohorts.

We conceived an analysis technique in which FCM data from 51 cellular parameters in an individual patient were compared non-parametrically to corresponding data from controls, generating centiles which could be depicted in heat maps. Heat maps could then be aligned within a kindred or clinical phenotype, allowing identification of consistent cellular trends. Here we demonstrate this technique by analyzing a cohort of patients with PID, including kindreds with known PID-associated mutations. The same technique should be applicable to guiding the search for new potentially pathogenic mutations uncovered in WGS screens.

## Methods

### Patient and Control Subjects

The cohort consisted of 77 control subjects and 199 patients with various immunological diagnoses, including autoimmunity (112), primary immunodeficiency conditions (57), oncology (21), neurological disease (5), sarcoidosis (3), and autoinflammatory conditions (1). Control subjects were aged between 20 and 72 years (median 40.5, mean ± SD: 40.8 ± 12.9) and were 64% female. We studied a subset of 22 patients from the primary immunodeficiency group who had primary antibody deficiency (PAD), 19 of whom met IUIS criteria for Common Variable Immunodeficiency (CVID) (2); the remaining 3 had specific antibody deficiency, 1 of whom was on infliximab (Supplementary Table 1). All PAD patients were on immunoglobulin replacement therapy and no other members of the PAD cohort were on immunosuppressives. PAD patients were aged between 10 and 72 years (median 36, mean ± SD: 38.3 ± 16.8) and were 55% female. See Supplementary Table 1 for details of patients reported in Figures 3–6.

Written informed consent was obtained as part of the Australian Point Mutation in Systemic Lupus Erythematosus study (APOSLE), the Centre for Personalized Immunology (CPI) program, the Healthy Blood Donors register and the Hematology Research Tissue Bank (The Canberra Hospital, Canberra, Australia). This study was carried out in accordance with the recommendations of the *National Statement on Ethical Conduct in Human Research (2007)*, National Health and Medical Research Council with written informed consent from all subjects. All subjects gave written informed consent in accordance with the Declaration of Helsinki. The protocol was approved by the Sydney Local Health District HREC at Concord Repatriation General Hospital, ACT Health HREC, ACT Hematology Research Tissue Bank Committee and Australian National University HREC.

### Cell Surface Staining Approach and Gating

We processed cells collected from the 276 subjects detailed above, many of whom had already undergone Whole Exome or Whole Genome sequencing under HREC-approved protocols, for flow cytometry. Blood was collected into ACD 9 ml collection tubes and processed within 24 h of collection. Peripheral blood mononuclear cells (PBMCs) were purified by layering blood over Ficoll-Paque, resuspended in RPMI, 10% DMSO and FCS, then stored in liquid nitrogen prior to analysis.

**Figure 1:**
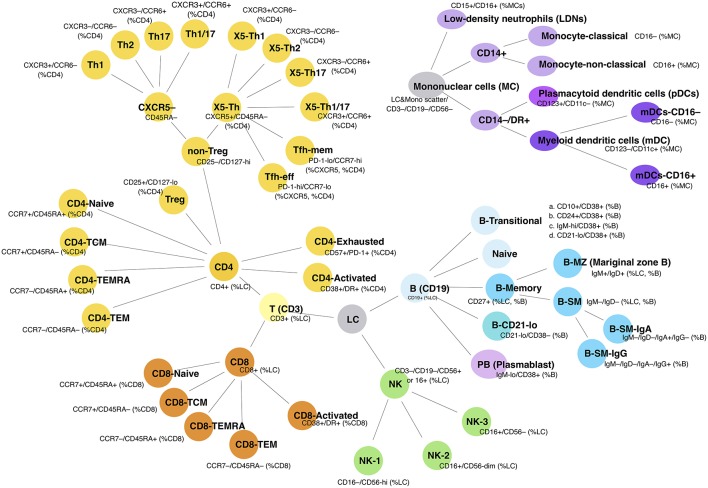
Cellular subpopulations as measured by FCM. Schematic shows the 54 separate cell populations determined using the four antibody panels (see section Methods). Lines show the relationship to parent populations.

Immunophenotyping of PBMCs was performed using 1 × 10^6^ cells for each staining panel. Cells were thawed at 37°C and washed with FACS buffer (PBS/2% bovine serum/0.05% sodium azide). Fc receptors were blocked using Human TruStain FcX (Cat #422302; Biolegend) and dead cells discrimination performed with LD Fixable Dead Cell stain (Cat #L34962; Invitrogen) according to the manufacturer's instructions. PBMCs were stained with four antibody cocktails (Supplementary Table 2) enabling identification of 54 cell parameters (Figure 1). Samples were fixed with eBioscience FoxP3 Fixation/Permeabilization reagent (Cat #00-5523-00; Invitrogen) according to the manufacturer's protocol. Flow cytometric acquisition was performed using a BD Fortessa FACS analyser at the Microscopy and Cytometry Resource Facility, John Curtin School of Medical Research and data analysis performed in Flowjo (v10; TreeStar). Repeated analysis of stored PBMCs from a single control was included with every FCM run, which allowed experimental variation in each parameter to be measured.

### Cell Populations and Methodological Aspects of FCM Analysis

The FCM analysis is shown in Supplementary Figure 1; initial gating was on viable singlet cells. Lymphocytes were gated by scatter; for non-lymphocyte populations, a broad leukocyte light scatter gate was used after excluding CD3, CD19, and CD56 expressing cells. Subpopulations were quantitated as a proportion of various parent populations, to generate up to 54 separate parameters per subject. Given varying approaches to defining transitional B cells, four commonly used gating strategies were initially applied: CD38+/CD10+, CD38+/CD24+, CD38+/IgM-hi, and CD38+/CD21-lo (Figure 1 and Supplementary Figure 1), although the latter strategy was abandoned because of its inclusion of circulating plasmablasts. Since the three remaining subsets were highly correlated (Supplementary Figure 2), we chose to use the CD38+/CD24+ proportion of B cells to represent the transitional cell population, leaving 51 total FCM parameters. NK cells, defined as CD3–/CD19– lymphocytes that expressed either CD16 or CD56, could be divided into three subpopulations (CD16–/CD56-hi, CD16+/CD56-dim, and CD16+/CD56–), and these were arbitrarily designated NK-1, -2, and -3, respectively (Figure 1 and Supplementary Figure 1). In the process of analysis, we found inconsistencies in CD4 T-helper (Th) chemokine receptor staining, such that values were censored when they were unable to clearly delineate four Th cell populations. We also observed that low density neutrophils appeared to accumulate with increasing time between collection and cryopreservation (data not shown).

In attempting to analyse memory T cell populations, we noted that some subjects over-expressed CD45RA, making precise gating impossible; these samples were censored for memory T cell parameters. This notable phenomenon reflects the action of a synonymous C > G variant at nucleotide position 77 in exon 4 (rs17612648) of the Protein tyrosine phosphatase receptor type C gene (*PTPRC*) that encodes the CD45 pan leukocyte receptor, that has previously been shown to interfere with CD45 splicing in T cells (3, 4). The variant allele, occurring in approximately 8% of individuals, acts in a dominant manner to maintain CD45RA expression on T cells following *in vitro* cell activation (5, 6). Although memory cell formation and function is not affected (5), T cells of individuals carrying this allele do not downregulate CD45RA. Since many subjects in our cohort had had genetic sequencing, we were able to identify seven individuals carrying the *PTPRC*^*C*77*G*^ allele who had also undergone immunophenotyping. Consistent with previous reports, we observed that all CD4+ and CD8+ T cells in these individuals expressed high levels of CD45RA (Supplementary Figure 3). The effect was more pronounced on CD8+ cells than CD4+ T cells, however a true CD45RA– population was not evident in either subset of T cell. Interestingly, we did not identify any CD45RA over-expressors in our healthy controls.

### Description of Normalization Algorithm/Software

**Figure 2:**
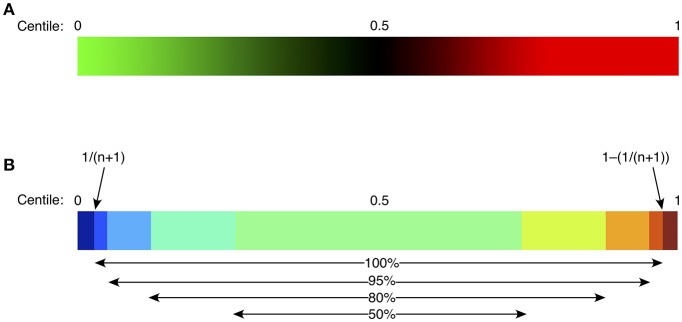
Heat mapping strategy for continuous green-black-red shading **(A)**, or discontinuous shading **(B)**. For continuous shading, colors vary continuously in shades of green (below control median), or red (above control median) with an intensity proportional to the difference from the median (0.5 centile). For discontinuous shading, colors vary stepwise as shown, based on the difference from the median, with the middle 50% of the control population in pale green, then progressively from light to dark blue (below control median), or yellow/orange/red/brown (above control median). Centiles for the control population are defined to vary from 1/(n+1) to 1–[1/(n+1)], depicted as “100%” in **(B)**, where n is the size of the control population; such an approach allows identification of “extreme” or unprecedented values, namely those lying outside the bounds of the control population. Other percentages represent the proportion of the control population included in each indicated color stratum.

Spreadsheet data for all FCM cellular populations from the study population were compared with the comparable data generated from controls. Bespoke software was written to analyse each subject parameter and to generate centiles by comparison with controls. To do this, the software sorted control values in ascending order and each one was allocated a centile value. The lowest value was allocated a centile of 1/(n+1), and the highest was 1–[1/(n+1)], where n is the number of values in the control cohort; this approach allowed extreme ‘unprecedented’ values, namely those which were never found in the control population, to be emphasized (Figure 2). The test parameter value was then compared with the sorted control values and attributed the corresponding centile (0–1), with further adjustment based on interpolation between the two neighboring control values. Centiles from each subject were then color coded in two ways: (i) continuously, where color intensity varied with deviation from the median (0.5 = black), values above the median depicted in intensity of red, and values below the median, in intensity of green; and (ii) discontinuously, where color strata represented variation from normal, differentiating the extreme 5, 10, or 25% in each direction; the central 50% of control values were represented as green (Figure 2).

### Statistics

All comparisons were performed in GraphPad Prism 8.0 for Mac, using Mann-Whitney non-parameteric comparison of non-paired values; *p*-values below 0.05 were considered significant.

### Cluster Analysis

To look for clusters of cellular changes within CVID patients, centile data from selected cellular parameters were generated. These data were loaded onto Morpheus (https://software.broadinstitute.org/morpheus/) and hierarchical clustering applied using 1-Pearson correlation.

## Results

### Non-parametric Heatmapping to Depict Cellular Changes in Patients With Primary Antibody Deficiency

**Figure 3:**
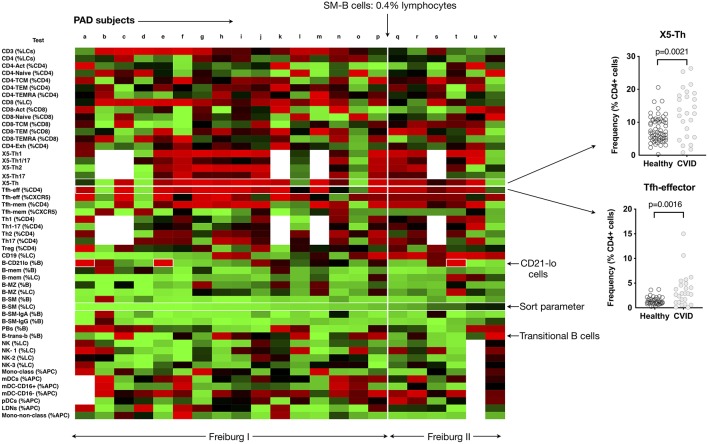
Heat mapping with continuous color shading (as depicted in Figure 2A) in PAD patients. Heat maps from individual patients (labeled a to v) are ordered from left to right based on increasing proportions of switched memory B cells (B-SM), with the cut-off of 0.4% of total lymphocytes shown. The expansion of CD21-lo B cells can be readily appreciated (labeled), as can the expansion in CXCR5+/CD45RA–CD4 T cells (X5-Th), and Tfh-effector cells and (as a percentage of CD4 T cells). Inset shows the comparisons between control values (*n* = 77) and PAD patients for X5-Th and Tfh-effector cells. Details of PAD patients presented in Supplementary Table 2.

Flow cytometry has an important role in understanding cellular changes in primary antibody deficiency disorders, particularly Common Variable Immunodeficiency (CVID). Sub-classifications have been based on differences in B-cell maturation patterns, the critical parameters including the proportions of total B cells, switched memory B cells (B-SM), CD21-lo B cells (sometimes referred to as “anergic” B cells) and transitional B cells (7, 8). We applied our analysis technique to our cohort of 22 patients with Primary Antibody Deficiency (PAD), 19 of whom met criteria for CVID (Supplementary Table 1), using heat mapping with continuous color shading (Figure 2A) to visually represent variations in the measured 51 cellular parameters, sorting patients on the basis of an ascending value for B-SM cells. This technique readily identified the known reduction in B-SM cells in CVID (Figure 3), but also demonstrated that this parameter was a continuous (rather than dichotomous) variable amongst PAD patients. Thus, whilst 16/22 (74%) subjects fulfilled objective criteria for “deficiency” of B-SM cells—values less or equal to the published cut-off of 0.4% of lymphocytes, also referred to as Freiberg I (9, 10)—the trend to SM-B cell reduction was evident in all but two patients (Figure 3, subjects “u” and “v”).

The reported expansion in CD21-lo cells could also readily be discerned from the PAD heat-map (Figure 3), particularly prominent in three patients (boxed, Figure 3), but was found in patients with “normal” as well as statistically deficient B-SM cells. Although our cohort was too small to make firm conclusions about clinical associations, we noted that 2 of the 3 patients with CD21-lo B cell expansions also had clinical lymphoproliferation and autoimmunity (Figure 3, subjects “e” and “t,” Supplementary Table 1), both reported associations (7, 11–13). A subset of patients with expansion of transitional B cells could also readily be appreciated (Figure 3) (8).

We could also demonstrate expansion of circulating CXCR5+/CD45RA− CD4+ T helper cells (here termed X5-Th) as a feature of PAD (Figure 3) (14, 15). Both the X5-Th cells and as well as the PD-1+/CD45RA-/CCR7-lo subpopulation (here termed Tfh-effector cells), a population that has been shown to correlate more closely with germinal center activity (16, 17), were significantly increased (*p* = 0.0021 and *p* = 0.0016, respectively). In contrast to previous reports (15), we found no correlation between the degree of SM-B cell depletion and the proportion of X5-Th cells (data not shown).

Thus, simultaneous representation of FCM parameters in a heat map, statistically normalized to a control population, can readily identify the known cellular patterns and trends in PAD patients, but also has the potential to facilitate discovery of new cellular changes not previously identified.

### Normalized Heat Mapping in Genetic Immunodeficiencies

Cellular changes might be noted in a proportion of patients with PAD, as demonstrated above, but no single change is characteristic. This is likely due to the fact that multiple gene mutations can result in the heterogeneous PAD clinical phenotype, with recent reports suggesting that up 30–40% of patients may have a monogenic disorder (18–21). Stratifying PIDs based on genetic mutation should result in more uniform cellular findings and reduce heterogeneity. Furthermore, since many of the identified causes are autosomal dominant with incomplete penetrance, cellular phenotyping might have the potential to distinguish the clinical phenotype of the mutation from healthy relatives with the same mutation. On the other hand, such changes might be missed if abnormalities are defined by and restricted to 95% confidence interval cut-offs. Using heat maps to depict variations from normal in patients with known mutations in *TNFRSF13B, CTLA4*, and *CARD11*, we asked whether we could identify trends within defined cellular populations that might be relevant to the genetic pathogenesis of their immunodeficiency.

#### TACI

Heterozygous and homozygous mutations in *TNFRSF13B* encoding the lymphocyte receptor TACI (transmembrane activator and calcium-modulating cyclophilin ligand interactor) are associated with CVID (22–24). Mutations affecting *TNFRSF13B* are found in 8% of CVID patients, however they are also found in 2% of the normal population (24), indicating that *TNFRSF13B* is a genetic risk factor for CVID, with disease expression presumably dependent on genetic modifiers or environmental triggers. Although *TNFRSF13B* is a highly polymorphic gene, two damaging variants resulting in protein mutations C104R and A181E (25) are most strongly associated with antibody failure (24, 26). We identified four patients with the A181E mutation in our CVID cohort, three heterozygous and one homozygous; we compared the cellular profiles of these four patients with two unaffected members of our cohort who had TACI mutations (one C104R, one A181E) but without antibody deficiency. Depicting the cellular changes as heatmaps, we looked for variations common to TACI mutants that were not present in unaffected individuals, and then statistically analyzed relevant parameters.

**Figure 4:**
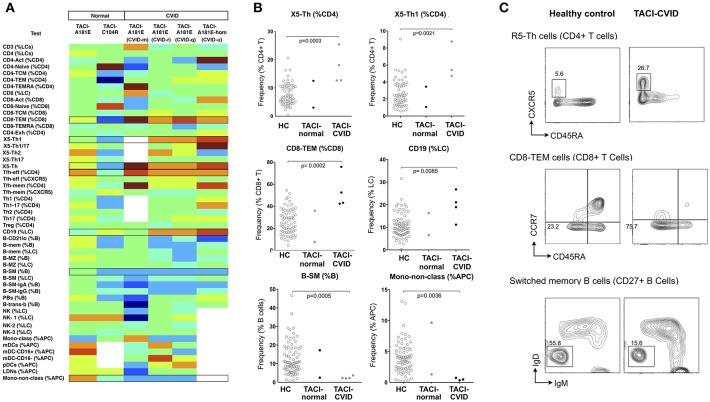
Analysis of cellular parameters in CVID patients carrying TACI variants in comparison to TACI mutant control subjects without CVID. **(A)** Heat mapping with discontinuous shading for four unrelated CVID patients carrying the TACI A181E allele, one homozygous, along with two healthy individuals with A181E, or C104R alleles. Boxes highlight parameters for which cellular changes differ in those with CVID compared with TACI controls. **(B)** Scatter plots showing raw values for populations identified in **(A)**, along with representative FCM contour plots of these critical parameters **(C)**. Raw and centile data is presented in Supplementary Table 3.

TACI mutant patients in our cohort showed consistently higher proportions of X5-Th cells, particularly their Th1 counterparts (X5-Th1), and Tfh-effector cells (Figure 4 and Supplementary Table 3), with the X5-Th cell expansion appearing even more prominently in TACI mutants than in the PAD cohort overall (Figure 3); we noted however that the difference found in the PAD cohort remained statistically significant after removal of the TACI mutants (data not shown). TACI mutants also showed expansion of CD8+ effector memory T cells, and total B cells, with reductions in B-SM cells and non-classical monocytes (Figure 4). Even though most of these changes did not lie outside the 95% confidence intervals (Supplementary Table 3), when analyzed as a group, the differences were significant (Figure 4B). Interestingly, we saw a similar increase in X5-Th cell cells in the patient homozygous for TACI mutation as in the heterozygotes, whereas it has been previously reported that only heterozygous TACI patients showed this expansion (27).

#### CTLA4 Patients

**Figure 5:**
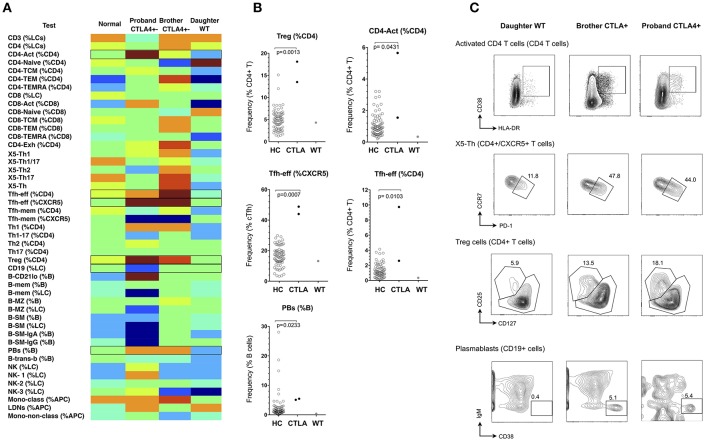
Analysis of cellular parameters in a CTLA4 haploinsufficient kindred. **(A)** Heat mapping with discontinuous shading showing changes in cell populations for the CTLA4 haploinsufficient proband with CVID, in comparison with the clinically normal brother with the CTLA4 mutation, the unaffected proband's adult daughter without CTLA4 mutation and the internal control. Boxes highlight parameters for which cellular changes differ between the two CTLA4 mutants, the unaffected subjects, and between the CTLA4 mutants with or without clinical expression. **(B)** Scatter plots showing raw values for populations identified in **(A)**, along with representative FCM contour plots of these critical parameters **(C)**. Raw and centile data is presented in Supplementary Table 3.

Heterozygosity for loss-of-function mutations in *CTLA4* gives rise to an autosomal dominant autoimmune lymphoproliferative syndrome (Type V MIM# 616100) with incomplete penetrance (28, 29). Clinical features are similar to CVID with an added propensity to autoimmunity. Reported cellular abnormalities have included expansion of CD21-lo B cells, reduction in naive T cells, over-expression of PD-1 by T-cells, and sometimes expansion of regulatory T (Treg) cells (28, 29). We studied a kindred with CTLA4 haploinsufficiency (heterozygous c.152_ins+GA), and generated normalized heat-maps of the heterozygous proband (profound hypogammaglobulinaemia, enteropathy and interstitial lung disease), the heterozygous brother (minimal clinical manifestations), and the unaffected adult daughter who did not carry the variant CTLA4 allele (Figure 5). From the resulting heatmap we observed that CTLA4 haploinsufficiency itself resulted in expansion of Treg cells, activated CD4+ cells, Tfh-effector cells (CCR7-lo/PD-1-hi, as a percentage of either CD4+ T cells, or CXCR5+ CD4+ T cells), and circulating plasmablasts, irrespective of the clinical phenotype (Figure 5 and Supplementary Table 3). In contrast, only the hypogammaglobulinaemic proband showed reduction of B cells (<1%).

#### CARD11

**Figure 6:**
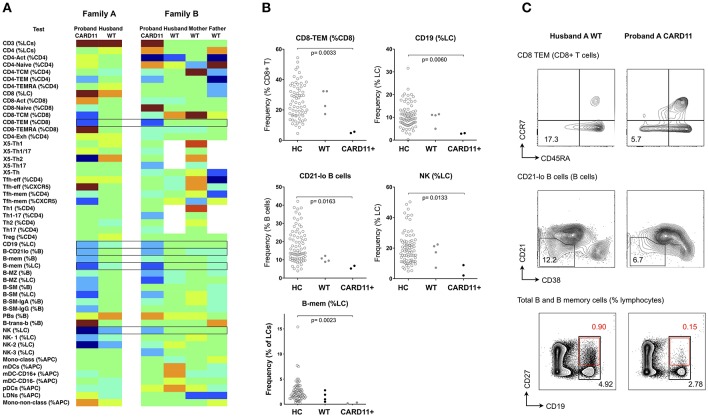
Analysis of cellular parameters in a CARD11 mutant kindred. **(A)** Heat mapping with discontinuous shading showing changes in cell populations for two unrelated patients with dominant negative CARD11 mutations, along with their relative(s) without mutation. Boxes highlight cellular changes common to the two CARD11 mutants, but differing from the family members. **(B)** Scatter plots showing raw values for populations identified in **(A)**, along with representative FCM contour plots of these critical parameters **(C)**; in the B-cell contour plot, black boxes and numbers refer to total CD19+ cells, and red boxes and numbers refer to CD19+/CD27+ memory B cells. Raw and centile data is presented in Supplementary Table 3.

CARD11 is a scaffold protein distal to antigen-receptor engagement in lymphocytes; dominant negative mutations give rise to a diverse clinical phenotype which includes atopic disease, autoimmunity and a combined immunodeficiency involving both B and T cell immunodeficiency (30). Mutations result in defects of T cell activation, NFκB activation and production of cytokines, such as IFN-γ, and IL-2 (20, 31), however changes in cellular phenotype are less established. In a recent multicenter study, we and others reported B-cell defects, including low total and memory B-cells, along with increased naïve and decreased memory T cells, however changes in peripheral lymphocytes differed considerably depending on the specific *CARD11* mutation (30). Using the same technique described above, we studied two unrelated kindreds with two different dominant negative CARD11 mutations (R47H (c.140G>A), and R974C (c.2920C>T), previously reported (30). In the two affected patients, there was reduction in CD8+ effector memory T cells, total NK cells and B cells (the latter two parameters giving rise to a relative increase in total T cell proportion), along with a reduction in the CD21-lo proportion of B cells (Figure 6 and Supplementary Table 3).

### Critical Cell Population Changes Determined by Ranking FCM Parameters by Laboratory and Disease Variation

We have demonstrated that cellular trends within a clinical phenotype or genotype can be readily depicted using our approach, but the inherent variability of FCM could challenge the reliability of the technique for general application. Such variability might relate to interlaboratory differences in monoclonal antibodies, their conjugate, the flow cytometer and its set-up, fluorescence compensation settings, and variations in subjective gating strategies between operators, but also to stochastic factors within the laboratory and inherent to the methodology itself. To measure inter-assay variation in our laboratory, we analyzed the same frozen cells from a single subject (collected on three separate occasions) in each of 28 runs, and measured the co-efficients of variation (CV) for the 54 original analysis parameters. Despite using the same monoclonal antibodies, conjugate, dilution, flow cytometer, operators (RT, AL) and gating review (DF), more than half of the parameters measured showed significant imprecision, with CV values above 30% for 36/54 parameters (Supplementary Figure 4). In the case of CVID, such laboratory variations could have significant implications for assessment, diagnosis and classification; considering only those parameters used for classification of CVID, we still found intra-laboratory CV values around 30% (Supplementary Figure 4), meaning that individual patients could vary in whether or not they fulfilled diagnostic criteria for CVID, or else be classified into different sub-groups at different timepoints, depending on random variations in the methodology alone.

Despite challenges posed by intra-laboratory variability, we asked whether parameters could be chosen that show more acceptable laboratory variation yet vary even more widely within patient populations. If so, realistically measurable cell parameters could be prioritized to ‘profile’ more reliably a specific immune disease phenotype (e.g., CVID, lupus, Sjogren's), or to assist in the diagnosis of immune disease, based on the hypothesis that there exist unique combinations of cellular changes characteristic to a disease or a disease subset.

**Figure 7:**
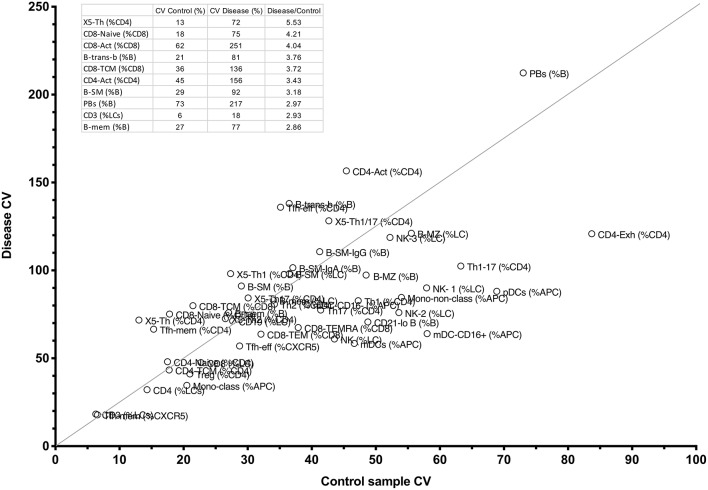
Comparison between CV percentages of 51 cellular parameters derived from the internal FCM control, and the same variation in the entire CPI cohort with widely varying immune conditions (see section Methods). The line shows the cut-off for the top 10 parameters with the highest ratio of disease-driven variation divided by internal variation, and these 10 parameters, and corresponding raw values appear in the table inset.

To identify such critical cell parameters, we plotted co-efficients of variation for the control sample (reflecting intra-laboratory variation) against variation for each test parameter in the entire disease cohort of 199 patients with miscellaneous conditions, including PID, autoimmunity, autoinflammatory disease, and cancer (see section Methods). By calculating the quotient of disease-based variation against inter-assay variation, we identified the top parameters that might be most useful for this purpose (Figure 7 and inset). Interestingly, X5-Th cells and their derivatives (Tfh-effector and Tfh-memory cells, X5-Th1, X5-Th1/17 cells, and X5-Th17 cells, all as %CD4 T cells), emerged as reproducible cell parameters that were at the same time most variable in disease; as these values all varied in parallel to the parent population (X5-Th cells), the latter parameter was deemed the most independent for cluster analysis (see below). Other critical parameters were naïve, activated and central memory CD8+ T cells, transitional B cells, memory B cells, SM-B cells, plasmablasts, activated CD4 cells, and total T cells (inset, Figure 7). On the other hand, the remaining traditional lymphocyte subsets frequently measured in the diagnosis of immune disease, namely CD4, CD8, CD19, and total NK cells, whilst reproducible methodologically, showed much less variation in disease, and hence might be less useful in practice than other cellular populations now quantifiable in the laboratory. Finally, when we restricted analysis of parameter variation to the PID patients alone, IgA-expressing switched memory B cells (B-SM-IgA (%B)) emerged as an important variable in this cohort, in addition to the those identified above (data not shown).

**Figure 8:**
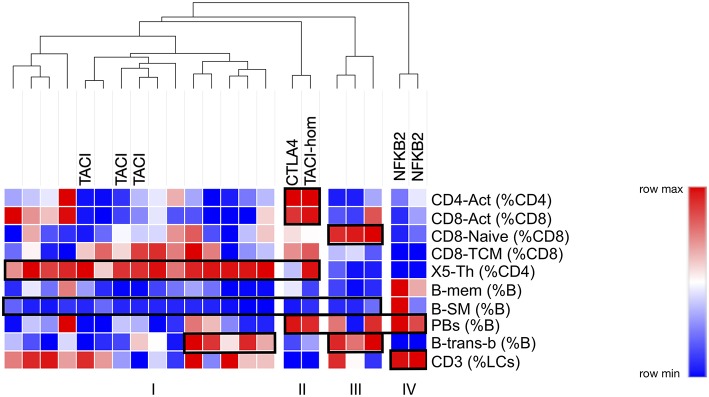
Cluster analysis of PAD patients based on centiles of the top 10 cellular parameters determined as outlined in the legend to Figure 7 (see text). Hereditary branches show relatedness in terms of cellular similarities, the major ones highlighted in boxes. Known genotypes are shown.

Finally, we asked the question as to whether restricting analysis to these critical 10 parameters might have the potential to stratify better PAD patients, perhaps giving clues to their genetic pathogenesis. By generating centiles for the 10 most discriminating parameters in our PAD cohort, and submitting them for cluster analysis, we were indeed able to identify a number of subgroups (Figure 8). The largest subgroup (I) was characterized by expansion of X5-Th cells with loss of B-SM cells; this subgroup included all TACI heterozygotes. Interestingly, the TACI homozygote clustered with the CTLA heterozygote, a group which were characterized by the above two characteristics along with T-cell (both CD4 and CD8) activation (Figure 8, subgroup II). A third subgroup lacked X5-Th cell expansion, but showed expansion of transitional B cells and naïve CD8 cells (Figure 8, subgroup III). Finally, the two patients with NFKB2 mutation were distinct from the remaining PAD patients, lacking X5-Th cell expansion, but with expansion of plasmablasts and decreased transitional B cells (subgroup IV). The addition of B-SM-IgA (%B) to the other 10 parameters did not change these subgroups, presumably because all but two of the PAD cohort were depleted of IgA-expressing switched memory B cells (data not shown); nevertheless, including this parameter may be important when analyzing PID cohorts not uniformly selected on the basis of antibody failure.

## Discussion

Flow cytometry facilitates rapid quantitation of multiple circulating white blood cell subpopulations, yet depicting variations in these parameters in disease, and associating such changes with corresponding genetic mutations, can be difficult. Here we report a non-parametric method for mapping variations in cellular subpopulations to facilitate detection of relevant cellular trends; raw patient values were converted into centiles based on comparison to controls, the centiles were mapped to color changes, and then the colors were assembled into a heat map. By aligning heat maps within a disease cohort (PAD, Figure 3), a genetic cohort (TACI, Figure 4) or within one or more genetic kindreds (CTLA4, Figure 5; CARD11, Figure 6), we have demonstrated how cellular changes can be readily identified, and specific candidate parameters chosen for definitive statistical analysis. For example, reported cellular changes associated with CVID were readily appreciated (Figure 3), and cellular fingerprints were successfully identified in the genetic cohorts or kindreds (Figures 4–6).

Our method of non-parametric heat mapping thus has the potential to detect more subtle, but nevertheless relevant and consistent, cellular changes than would be detected by applying traditional “normal ranges” based on the control mean plus or minus two standard deviations (parametric distributions), or 95% confidence intervals (non-parametric distributions). Whilst reference ranges are important for an individual patient, when studying cohorts of patients or kindreds, variations in cellular parameters, either increased or decreased, might show consistent, and pathogenically relevant trends even though individual values might lie within the target range. For example, most of the changes we found in our genetic subgroups were statistically significant despite individually having centiles between 0.025 and 0.975 (Supplementary Table 3), which would otherwise represent the non-parametric normal range. Furthermore, when searching out real alterations in cell composition that are dependent on the possible effects of subtle gene changes, one cannot assume that the biological effect on a given cell population will be so extreme that they can be categorized into “expanded” vs. “deficient,” based on a defined 95% confidence interval alone.

Defining cellular changes in immune disease could therefore generate hypotheses regarding the pathogenesis of genetic immunodeficiencies. We noted, for example, the expansion of X5-Th cells in CVID patients, particularly in those with *TNFSF13B* mutations. It is perhaps counter-intuitive that patients with antibody deficiency should have increased X5-Th cells (commonly referred to as circulating T-follicular helper cells, cTfh), a phenotype that has been frequently associated with autoimmunity (32). However, studies in TACI deficient mice have shown that expansion of Tfh cells is extrinsically regulated by increased expression of ICOSL on B cells, and that antibody deficiency results from the important role that TACI plays in promoting plasma cell survival (33). Also, whether expansion of X5-Th cells reflects heightened Tfh cell activity or numbers in secondary lymphoid tissues of CVID patients remains unknown. Furthermore, Tfh cells are B cell dependent, and have been shown to be deficient in patients who lack B cells as a result of BTK deficiency (34); this phenomenon was observed in our NFKB2 patients, who also exhibited profound B cell deficiency (subjects “b” and “d,” Figure 3). Given the association of cTfh cells with autoimmunity, it is tempting to speculate that this change might also play a pathogenic role in the increase propensity to autoimmunity in CVID patients with TACI-deficiency (35). However, it cannot be assumed that an increase in cTfh cell numbers would necessarily correlate with an increase in cTfh cell function; analysis of cTfh cells from a range of different genetic PIDs has revealed that mutations can impact both the quality and quantity of cTfh cells (36), and thus it is relevant to ask whether the increased frequency of cTfh cells in TACI-mutant CVID patients also correlates with changes in function.

We also observed increased Tfh-effector cells and plasmablasts in CTLA4 haploinsufficient subjects (Figure 5), which has not previously been reported. Whilst our numbers are small and require further validation in additional kindreds, overactivity of Tfh-effector cells would be consistent with the spontaneous development of germinal centers in CTLA4-deficient mice (37) and might suggest that in addition to the defect in negative regulation due to reduced expression of CTLA4, Tfh effector cells might drive increased germinal centre output and possibly contribute to the increased propensity toward autoimmunity in these patients. The similarity in cellular phenotype in the patient and her unaffected brother does raise questions as to the pathogenesis of the clinical expression in CTLA4 haploinsufficiency, and whether these patients are ‘primed’ for susceptibility to environmental factors that might be responsible for driving the antibody deficiency and, in this patient, B-cell loss.

Non-parametric heat mapping might also facilitate the search for genetic explanations of immune disease, based on the assumption that a mutation or polymorphism under study is pathogenically relevant and alters cellular subpopulations in the blood. To do this, heat maps from members of a kindred can be assembled, and cell parameters that associate with specific genetic changes identified for further analysis. Should such cellular fingerprints prove to be characteristic of a particular genotype, diagnosis of immunodeficiency diseases could be facilitated by aligning the cellular heatmap of a new patient with an immunodeficiency (or other immune) condition with the heatmaps of other patients with established clinical or genetic diagnoses, similarities in critical parameters prompting a provisional diagnosis prior to confirmatory testing. For example, in the CTLA4 kindred (Figure 5), the lack of characteristic cellular changes in the daughter suggested her WT genotype well-before the CTLA4 sequencing results were known. Similarly, the clustering of the two NFKB2-deficient patients together on the basis of 10 critical FCM parameters (Figure 8) implies that this approach could also be used to prompt a genetic diagnosis based on cellular phenotype alone.

Analysis of FCM data in this way may however be limited by the wide inter-assay variation that seems intrinsic to FCM as a technique; despite careful control of methodological conditions, we noted high CV values for most cellular parameters measured (Supplementary Figure 4). It should be evident that such variation challenges the wide-spread application of defined cut-offs, for example in CVID, since the same patient may change their classification or ability to fulfill diagnostic criteria based on random laboratory variables alone. We have addressed this laboratory variation in two ways. Firstly, we have depicted variation using normalized heat mapping such that trends can be detected even if they do not lie outside reference ranges. Using PAD as an example, we were able to demonstrate known trends without the need to define “normal” vs. “abnormal” (Figure 3). Secondly, we selected critical parameters that were both reproducible in the laboratory and also most variable in disease, based on the observation that disease-related cellular variation always exceeded intra-laboratory variations (Figure 7), and that cellular parameters could be ranked in terms of their utility for such profiling. We demonstrated this approach in PAD, where clusters of changes could be identified based on a selection of 10 of the most discriminating cellular parameters, and in some cases could be characteristic of a particularly genotype (NFKB2, Figure 8, subgroup IV). A similar approach could be used to look for critical commonalities within a heterogeneous immunological disease (e.g., lupus, Sjögren's), carefully selecting or weighting sorting parameters based on both their laboratory reproducibility and simultaneously their greater variation in immunological disease.

There were a few limitations to our study. Numbers within each study population or kindred were relatively small, and it would be important to apply our methodology to larger genetic cohorts. We stress however that the aim of our study was never to develop a new classification system for CVID, nor to provide definitive data on the cellular phenotypes of the genetic PIDs studies, as our relatively small numbers would never have had the power to replicate the findings of more substantial, multicenter cohorts such as those reported in the Freiburg or Euroclass studies (8, 9), particularly in regard to clinical correlations. Nevertheless, our technique of normalizing patient data to control samples may readily be applied to such larger cohorts, and that careful selection of differentiating parameters (Figure 8) might facilitate discovery of clustering algorithms that more closely correlate with the expanding array of genetic changes associated with immunologic disease.

Given the methodological variability in the FCM technique itself, it was also difficult to exclude that variations in specific cellular parameters in our cohorts might have arisen through such random variation alone. Nevertheless, the statistical comparisons we did employ were often highly significant, with *p*-values < 0.01. Since many of our cellular parameters were interdependent, adjustment for multiple comparisons was not feasible, however the False Discovery Rate when comparing 50 parameters would predict that <1 parameter should have *p-*value ≤ 0.01 by chance alone; given the number of cellular differences we were able to identify, often with *p*-values below 0.01, we believe that the changes we noted were not only real, but also afforded credence to the techniques used to identify them. Our technique might be less practical however in pediatric settings, given the need for reliable age-dependent control reference values. Some parameters, such as low-density neutrophils, were too methodologically variable to be useful, and few of the myeloid markers emerged as useful candidate parameters, perhaps expected given the focus here on “lymphocyte-centric” immunodeficiency diseases; such parameters might play a role in other (perhaps autoimmune) conditions where innate mechanisms are more prominent pathogenically. Despite these limitations, we were able to fulfill the main aim of our study namely to demonstrate an approach to visually depict trends within small cohorts, trends which can then be used to generate hypotheses for confirmation in larger groups.

Here we illustrate the use of non-parametric normalized heat mapping of FCM parameters to represent changes in cellular parameters in a phenotype or genotype. By relinquishing reliance on reference intervals, this technique can discern trends within a disease or kindred, possibly improve diagnosis or classification, and refine understanding of the pathogenic relevance of gene mutations in established and emerging PID.

## Data Availability

All datasets generated for this study are included in the manuscript and/or the Supplementary Files.

## Ethics Statement

This study was carried out in accordance with the recommendations of the National Statement on Ethical Conduct in Human Research (2007), National Health and Medical Research Council with written informed consent from all subjects. All subjects gave written informed consent in accordance with the Declaration of Helsinki. The protocol was approved by the Sydney Local Health District HREC at Concord Repatriation General Hospital, ACT Health HREC, ACT Hematology Research Tissue Bank Committee and Australian National University HREC.

## Author Contributions

DF and JE designed the study, analyzed data, and wrote the manuscript. RT, AL, AC, and RC performed experiments. SJ, DT, DF, and MC recruited patients and blood samples. A-MH and AW recruited patients and healthy controls and maintained clinical data. MC and CV provided intellectual input to the study design and manuscript.

### Conflict of Interest Statement

The authors declare that the research was conducted in the absence of any commercial or financial relationships that could be construed as a potential conflict of interest.

## Supplementary Material

Supplementary Figure 1 Immunophenotyping gating strategies. Flow cytometry plots showing the gating strategy employed to differentiate and quantify each of the 54 cell parameters. Plots are pre-gated on live cells or lymphocytes (as indicated) after removal of doublets. Four antibody FACS panels were used covering T cells **(A,B)**, B cells **(C)**, and myeloid/NK cells **(D)**. Cell populations are named on final gate. Markers used to define each gate are indicated. Arrows between plots indicate sub-gating.

Supplementary Figure 2 Correlation between values derived from 3 different transitional gating strategies (see section Methods). Scatter plots show the correlation between the frequency of transitional B cell populations in all analyzed patients and controls, as defined by either CD38+/CD24+ B cells (“Trans-b”) as the independent variable, in comparison to CD38+/CD10+ B cells (“Trans-a”) or CD38+/IgM-hi B cells (“Trans-c”).

Supplementary Figure 3 T cell memory subpopulation gating demonstrating CD45RA over-expression in an individual bearing the variant PTPRC G77 allele (bottom) compared to an individual with the wild type allele (top). Gating on CD4 (left) or CD8 T cells (right).

Supplementary Figure 4 Coefficients of Variation (CV) for all 54 FCM parameters. Values above 30% were considered to show significant imprecision and are shaded.

Supplementary Table 1 Details of the PID patients in the four cohorts analyzed in the cited figures.

Supplementary Table 2 Reagents used for staining cells for flow cytometry, for the four separate panels.

Supplementary Table 3 Raw percentages and derived centiles for each of the FCM parameters from subjects whose corresponding heatmaps are presented in Figures 4–6 (Cent. = centiles).
