# Supplementary figures and images for: Non-parametric Heat Map Representation of Flow Cytometry Data: Identifying Cellular Changes Associated With Genetic Immunodeficiency Disorders
Source: Front Immunol. 2019 Sep 11;10:2134. doi: 10.3389/fimmu.2019.02134 (PMC6749093; doi:10.3389/fimmu.2019.02134)

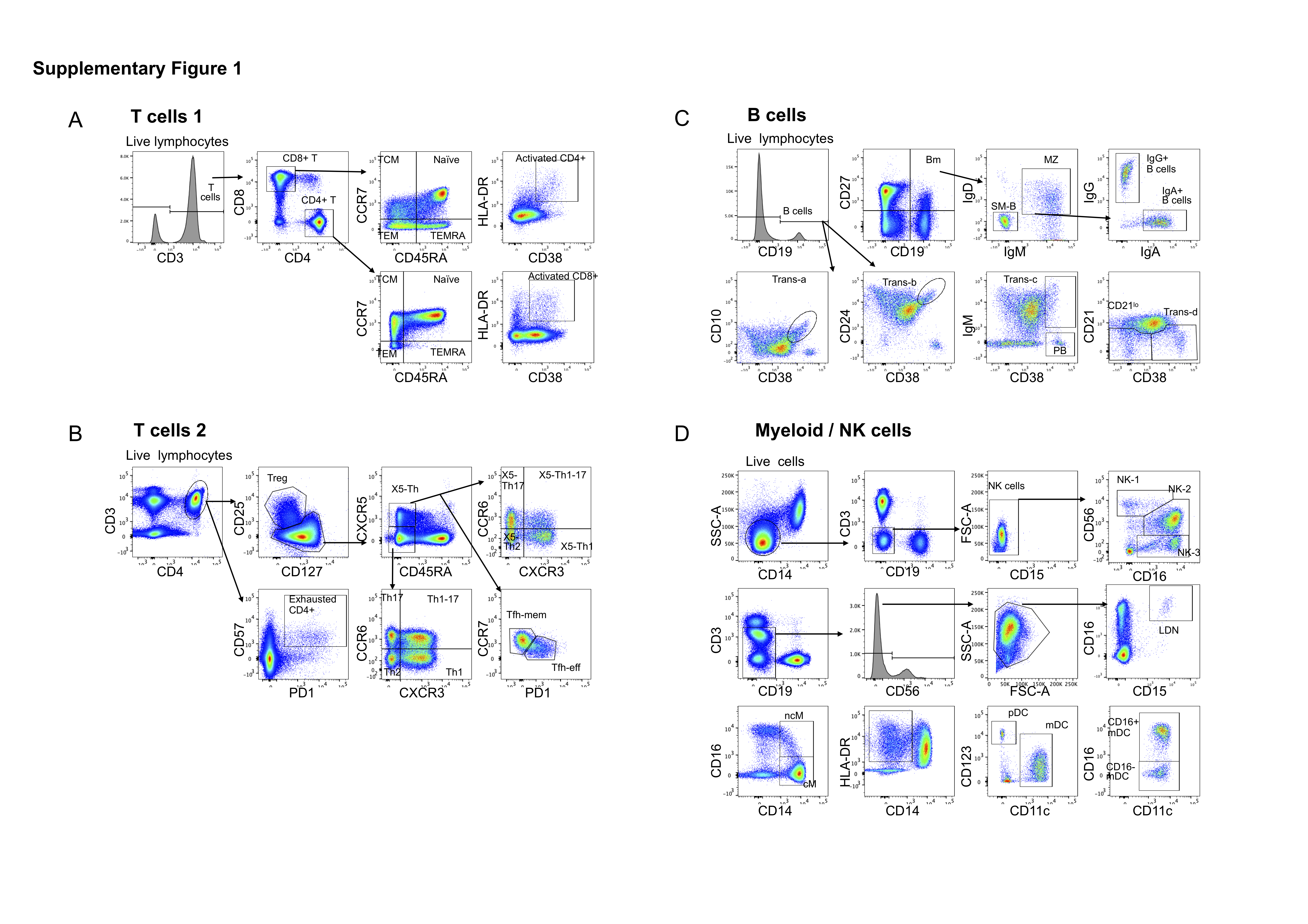

Supplement: Supplementary Figure 1 — Immunophenotyping gating strategies. Flow cytometry plots showing the gating strategy employed to differentiate and quantify each of the 54 cell parameters. Plots are pre-gated on live cells or lymphocytes (as indicated) after removal of doublets. Four antibody FACS panels were used covering T cells (A,B), B cells (C), and myeloid/NK cells (D). Cell populations are named on final gate. Markers used to define each gate are indicated. Arrows between plots indicate sub-gating. [file Image_1.TIF]

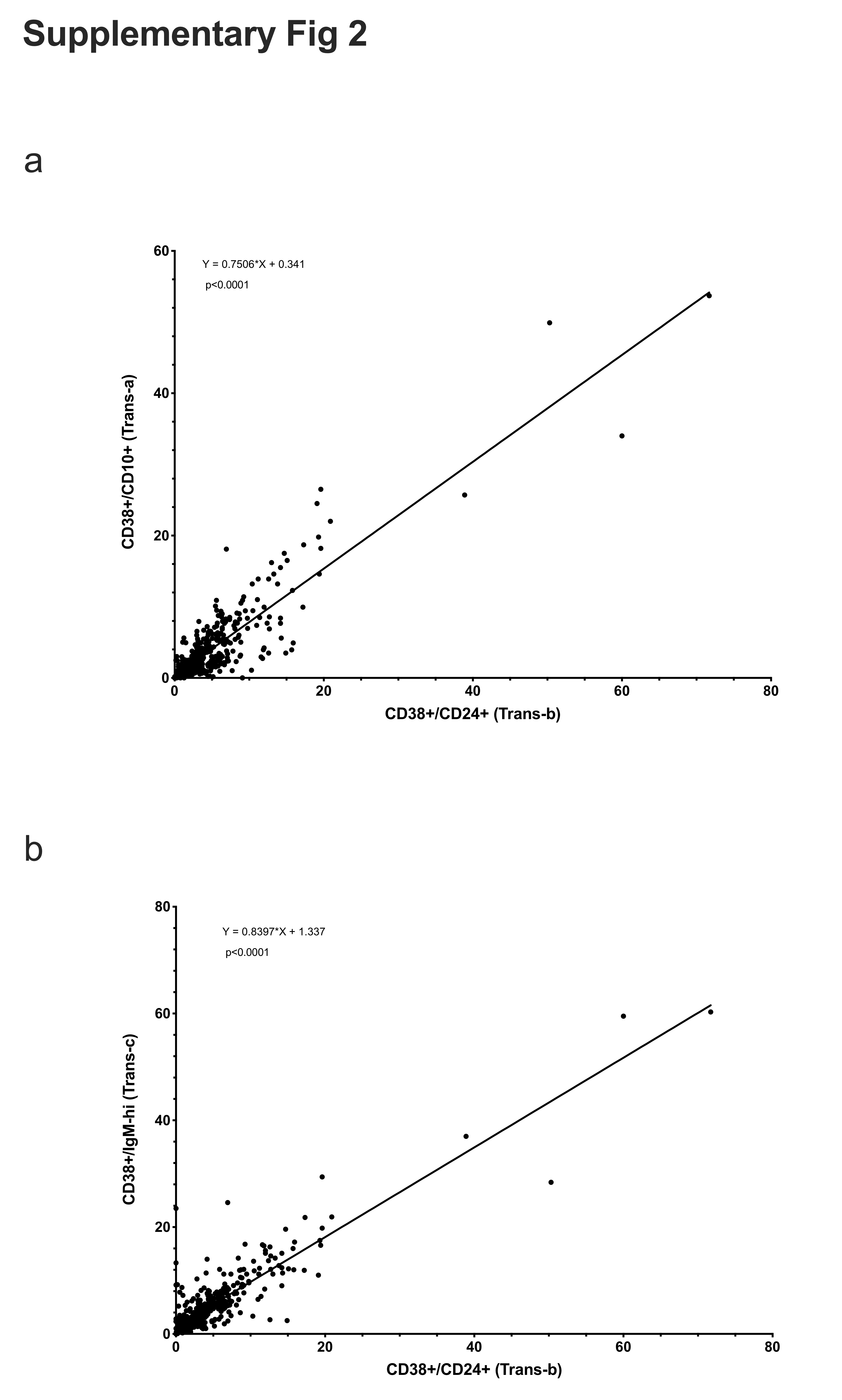

Supplement: Supplementary Figure 2 — Correlation between values derived from 3 different transitional gating strategies (see section Methods). Scatter plots show the correlation between the frequency of transitional B cell populations in all analyzed patients and controls, as defined by either CD38+/CD24+ B cells (“Trans-b”) as the independent variable, in comparison to CD38+/CD10+ B cells (“Trans-a”) or CD38+/IgM-hi B cells (“Trans-c”). [file Image_2.TIF]

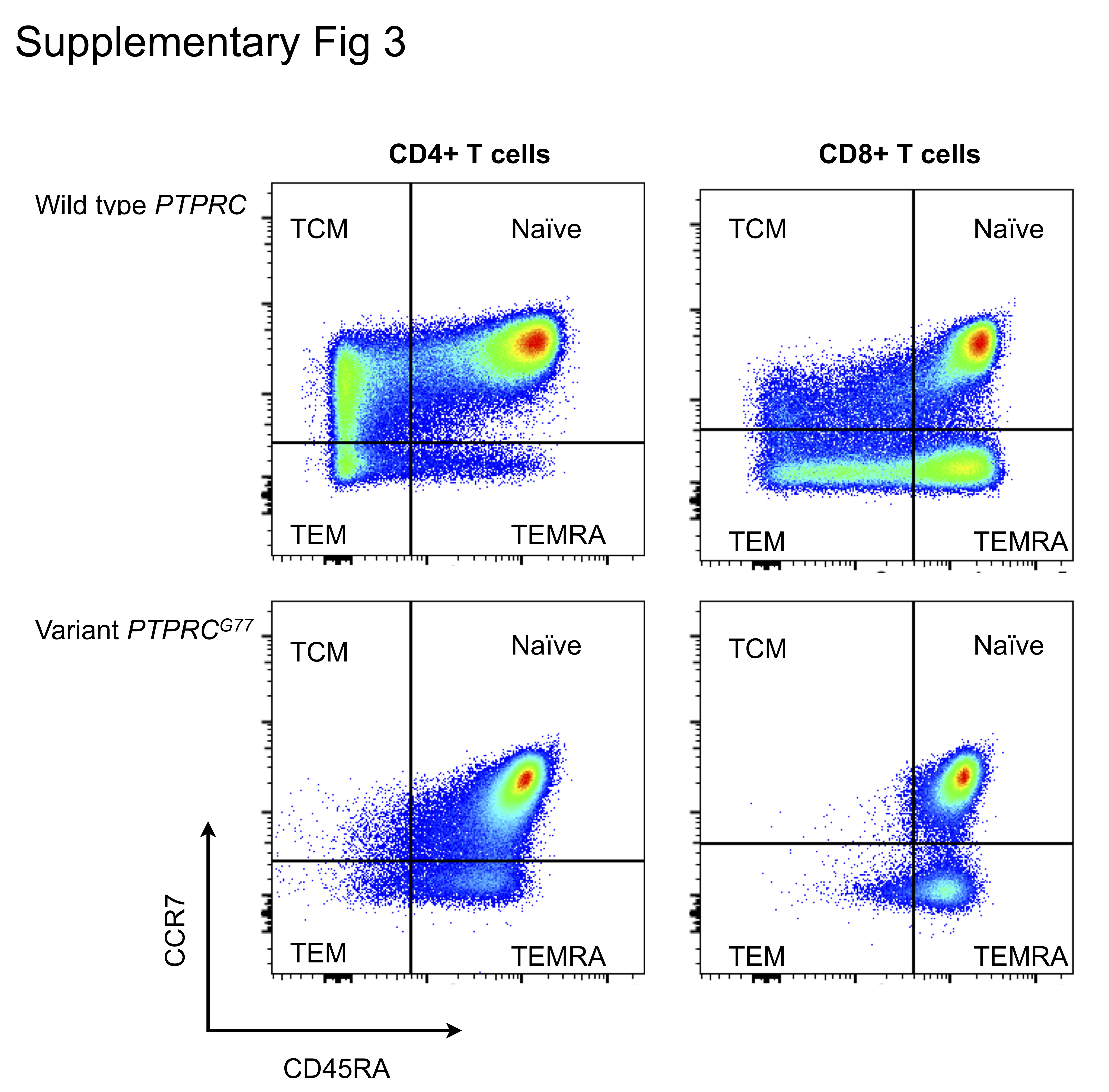

Supplement: Supplementary Figure 3 — T cell memory subpopulation gating demonstrating CD45RA over-expression in an individual bearing the variant PTPRC G77 allele (bottom) compared to an individual with the wild type allele (top). Gating on CD4 (left) or CD8 T cells (right). [file Image_3.TIF]

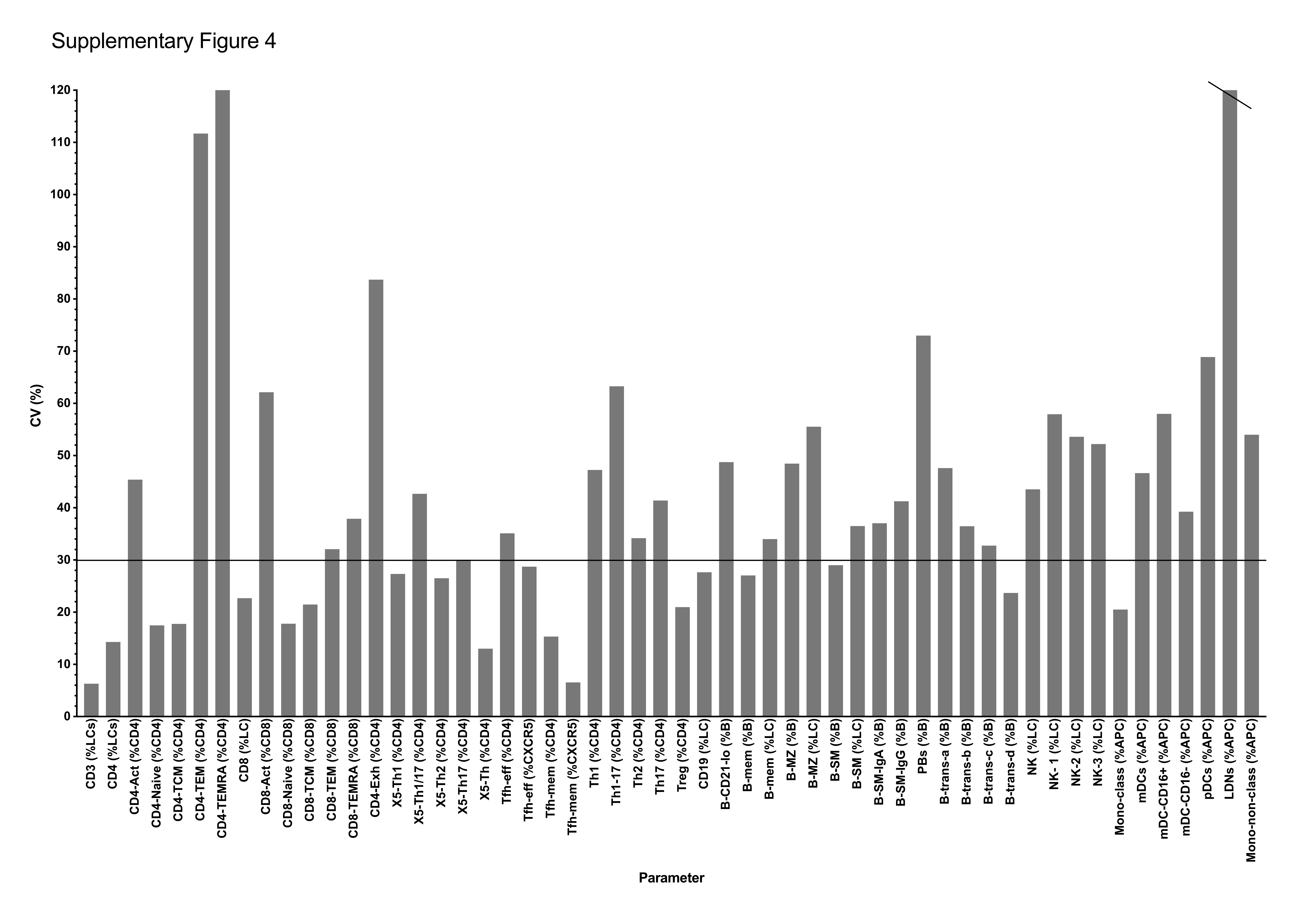

Supplement: Supplementary Figure 4 — Coefficients of Variation (CV) for all 54 FCM parameters. Values above 30% were considered to show significant imprecision and are shaded. [file Image_4.TIFF]
