# Supplementary material for: Non-parametric Heat Map Representation of Flow Cytometry Data: Identifying Cellular Changes Associated With Genetic Immunodeficiency Disorders
Source: Front Immunol. 2019 Sep 11;10:2134. doi: 10.3389/fimmu.2019.02134 (PMC6749093; doi:10.3389/fimmu.2019.02134)
Supplement: Supplementary Table 1 — Details of the PID patients in the four cohorts analyzed in the cited figures. [file Data_Sheet_1.PDF]

Supplementary Table 1

Referenced in Figure(s)	Sex	Age	Diagnosis	Gene	Mutation	Zygosity	Relationship	Lymphoproliferation	Granulomas	Autoimmunity	Other clinical manifestations
Fig 3: PAD - g	M	29	CVID					no	no	no	
Fig 3: PAD - m; Fig 6	F	42	CVID	TNFRSF13B	A181E	Heterozygous		no	no	no	
Fig 3: PAD - s	F	61	CVID					no	no	yes	Arthritis, Thrombocytopenia
Fig 3: PAD - f	M	47	SAD					no	no	no	
Fig 3: PAD - n	F	72	CVID					no	no	no	
Fig 3: PAD - c	M	52	CVID					yes	no	yes	Splenomegaly, Alopecia universalis
Fig 3: PAD - p	M	60	CVID					no	no	no	
Fig 3: PAD - r; Fig 6	F	22	CVID	TNFRSF13B	A181E	Heterozygous		no	no	no	
Fig 3: PAD - q; Fig 6	M	25	CVID	TNFRSF13B	A181E	Heterozygous		no	no	no	
Fig 3: PAD - e	M	37	CVID					yes	no	yes	Arthritis,Splenomegaly
Fig 3: PAD - k	F	43	CVID					no	no	no	
Fig 3: PAD - l	F	20	SAD					no	no	yes	Infliximab therapy for inflammatory bowel disease
Fig 3: PAD - t	M	26	CVID					yes	yes	no	Splenomegaly, Pulmonary eosinophilic granuloma
Fig 3: PAD - i	F	26	CVID					no	no	yes	Arthritis
Fig 3: PAD - j	M	40	CVID					no	no	no	
Fig 3: PAD - h	F	30	CVID					no	no	no	
Fig 3: PAD - b	F	60	CVID	NFKB2	D865G			no	no	no	
Fig 3: PAD - d	M	31	CVID	NFKB2	D865G			no	no	no	
Fig 3: PAD - v	M	10	CVID					no	no	no	Haemophagocytic lymphohistiocytosis
Fig 3: PAD - u; Fig 6	F	35	CVID	TNFRSF13B	A181E	Homozygous		no	no	no	
Fig 3: PAD - o	F	16	SAD					no	no	no	
Fig 3: CVID - a; Fig 7, Proband	F	59	PAD	CTLA4	c.151+GA	Heterozygous	Proband	no	no	yes	Arthritis, interstitial lung disease, enteropathy
Fig 5, Brother	M	62	Unaffected	CTLA4	c.151+GA	Heterozygous	Brother				
Fig 5, Daughter	F	30	Unaffected				Daughter				
Fig 4	F	45	Normal	TNFRSF13B	A181E						
Fig 4	F	28	Normal	TNFRSF13B	C104R		Daughter of deceased CVID patient				
Fig 6, Family A	F	40	CID	CARD11	R47H	Heterozygous, dominant negative	Proband				
Fig 6, Family A	M	41	Normal				Husband				
Fig 6, Family B	F	34	CID	CARD11	R974C	Heterozygous, weak dominant negative	Proband				
Fig 6, Family B	M	34	Normal				Husband				
Fig 6, Family B	F	57	Normal				Mother				
Fig 6, Family B	M	57	Normal				Father				
