# Supplementary material for: Non-parametric Heat Map Representation of Flow Cytometry Data: Identifying Cellular Changes Associated With Genetic Immunodeficiency Disorders
Source: Front Immunol. 2019 Sep 11;10:2134. doi: 10.3389/fimmu.2019.02134 (PMC6749093; doi:10.3389/fimmu.2019.02134)
Supplement: Supplementary Table 2 — Reagents used for staining cells for flow cytometry, for the four separate panels. [file Data_Sheet_2.PDF]

Supplementary Table 2

Stain	Monoclonal	Cat#	Clone	Supplier
T cells 1	CD3 Pacific Blue	558117	UCHT1	BD
	HLA-DR-BV510	307646	L243	Biolegend
	CCR7-Al488	353206	G043H7	Biolegend
	CD4-PerCP-Cy5.5	560650	RPAT4	BD
	CD45RA PE-Cy7	25-0458-73	HI100	eBioscience
	CD38 APC	A60792	LS198-4-3	Beckman Coulter
	CD8 APC -EF-780	47-0087-41	SK1	eBioscience
T cells 2	CD3 Pacific Blue	558117	UCHT1	BD
	CCR6-BV510	353424	G034E3	Biolegend
	CD127 -BV650	351326	AO19D5	Biolegend
	CD25 -BV605	302632	BC96	Biolegend
	CCR7-Al488	353206	G043H7	Biolegend
	CD4-PerCP-Cy5.5	560650	RPAT4	BD
	CXCR3-PE	353706	G025H7	Biolegend
	CD57-PE/Dazzle	359620	HNK-1	Biolegend
	CD45RA PE-Cy7	25-0458-73	HI100	eBioscience
	CXCR5-AF647	558113	RF8B2	BD
	CD279 (PD-1) Bi	13-2799	eBioJ105	eBioscience
	Streptavidin APC-eFluor® 780	47-4317		eBioscience
B cells	IgM EF450	48-9998	SADA4	eBioscience
	IgD-BV510	348220	IA6-2	Biolegend
	CD24 - BV 605	311124	MLS	Biolegend
	CD19 - BV 650	302238	HIB19	Biolegend
	CD38 PerCP-Cy5.5	551400	HIT2	BD
	IgA-PE	130-093-128	IS11-8E10	Miltenyi Biotech
	CD10-PE-CF594	562396	HI10a	BD
	IgG PE-Cy7	561298	G18-145	BD
	CD21-APC	559867	B-ly4	BD
	CD27 APC-EF-780	47-0279	O323	eBioscience
Myelod/NK cells	CD3 Pacific Blue	558117	UCHT1	BD
	HLA-DR-BV510	307646	L243	Biolegend
	CD19 - BV 650	302238	HIB19	Biolegend
	CD14-PerCP	340585	23-3781-00	BD
	CD123-PE	554529	7G3	BD
	CD56 PE-Cy7	335791	NCAM16.2	BD
	CD16-APC-H7	560195	3G8	BD
	CD11c-APC	559877	B-ly6	BD
