# Supplementary material for: Non-parametric Heat Map Representation of Flow Cytometry Data: Identifying Cellular Changes Associated With Genetic Immunodeficiency Disorders
Source: Front Immunol. 2019 Sep 11;10:2134. doi: 10.3389/fimmu.2019.02134 (PMC6749093; doi:10.3389/fimmu.2019.02134)
Supplement: Supplementary Table 3 — Raw percentages and derived centiles for each of the FCM parameters from subjects whose corresponding heatmaps are presented in Figures 4–6 (Cent. = centiles). [file Data_Sheet_3.PDF]

Supplementary Table 3

TACI Subjects

	Normal-TACI-A181E		Normal-TACI-C104R		CVID-TACI-A181E		CVID-TACI-A181E		CVID-TACI-A181E		CVID-TACI-A181E-hom	
	Raw	Cent.	Raw	Cent.	Raw	Cent.	Raw	Cent.	Raw	Cent.	Raw	Cent.
**CD3 (%LCs)**	69.8	0.57	61.7	0.29	80.5	0.94	57.4	0.2	66.2	0.45	57.6	0.21
**CD4 (%LCs)**	48	0.79	36.3	0.45	33.1	0.35	35.9	0.44	38.4	0.48	30.2	0.28
**CD4-Act (%CD4)**	0.46	0.14	0.71	0.33	0.28	0.03	0.75	0.36	0.43	0.09	4.52	1
**CD4-Naive (%CD4)**	39.3	0.33	85.7	1	7.89	0.02	49.5	0.49	46.2	0.46	21.7	0.09
**CD4-TCM (%CD4)**	48	0.79	12.2	0.04	27.5	0.24	40.8	0.64	37.3	0.59	49	0.79
**CD4-TEM (%CD4)**	12.1	0.65	1.68	0	14.3	0.73	8.6	0.45	15.7	0.76	28.4	0.9
**CD4-TEMRA (%CD4)**	0.6	0.34	0.49	0.23	50.4	1	1.05	0.52	0.74	0.43	0.89	0.47
**CD8 (%LC)**	18.9	0.42	16.3	0.32	41.9	0.97	14.2	0.21	21.2	0.5	18.4	0.4
**CD8-Act (%CD8)**	1.67	0.59	0.57	0.16	0.34	0.03	0.86	0.27	0.5	0.12	12	0.98
**CD8-Naive (%CD8)**	41.2	0.6	72.3	0.98	2.66	0.03	26.4	0.41	32	0.49	32.7	0.51
**CD8-TCM (%CD8)**	13	0.71	6.3	0.24	10.9	0.58	16.2	0.85	10.1	0.55	14.9	0.79
**CD8-TEM (%CD8)**	36	0.78	7.56	0.03	75.8	1	43.1	0.92	52.5	0.98	42.3	0.92
**CD8-TEMRA (%CD8)**	9.8	0.15	13.9	0.3	10.7	0.17	14.2	0.31	5.44	0.05	10.1	0.15
**CD4-Exh (%CD4)**	0.34	0.38	0.12	0.17	0.42	0.43	0.35	0.39	0.86	0.62	1.86	0.83
**X5-Th1 (%CD4)**	3.45	0.67	1.07	0.1	-	-	4.73	0.91	5.41	0.95	8.76	0.98
**X5-Th1/17 (%CD4)**	1.56	0.83	0.3	0.25	-	-	1.02	0.65	1.71	0.86	7.41	1
**X5-Th2 (%CD4)**	4.02	0.91	1.01	0.07	-	-	4.9	0.92	3.4	0.85	0.83	0.03
**X5-Th17 (%CD4)**	3.5	0.89	0.65	0.24	-	-	2.08	0.68	2.12	0.69	1.08	0.29
**X5-Th (%CD4)**	12.5	0.9	3.04	0.06	25.4	1	12.7	0.92	12.6	0.91	18.1	0.98
**Tfh-eff (%CD4)**	2.85	0.94	0.39	0.17	4.1	0.99	2.91	0.94	2.93	0.94	2.71	0.92
**Tfh-eff (%CXCR5)**	22.7	0.8	12.8	0.28	16.2	0.49	22.8	0.8	23.1	0.8	15	0.43
**Tfh-mem (%CD4)**	9.4	0.88	2.61	0.13	20.7	1	9.67	0.9	9.62	0.9	14.8	0.98
**Tfh-mem (%CXCR5)**	75	0.2	85.9	0.75	81.6	0.43	76	0.2	76.1	0.2	82.1	0.49
**Th1 (%CD4)**	9.29	0.44	3.29	0.04	-	-	12.1	0.66	9.77	0.51	19.2	0.88
**Th1-17 (%CD4)**	14.8	0.83	1.37	0.08	-	-	6.49	0.52	10.3	0.69	21.3	0.96
**Th2 (%CD4)**	10.7	0.71	6.8	0.4	-	-	11.9	0.74	6.45	0.35	3.94	0.12
**Th17 (%CD4)**	7.68	0.83	1.77	0.17	-	-	5.49	0.66	7.42	0.81	8.62	0.89
**Treg (%CD4)**	4.09	0.37	4.83	0.5	4.61	0.45	2.74	0.18	5.85	0.71	4.3	0.4
**CD19 (%LC)**	6.56	0.18	16.3	0.82	11.2	0.62	21.1	0.97	18.9	0.91	26.8	0.98
**B-CD21lo (%B)**	28.4	0.85	5.8	0.06	16.3	0.56	7.6	0.09	8.61	0.11	4.37	0.02
**B-mem (%B)**	30.5	0.76	8.69	0.09	13.4	0.28	4.3	0.03	3.94	0.03	10.2	0.13
**B-mem (%LC)**	2	0.49	1.42	0.28	1.5	0.33	0.91	0.13	0.74	0.09	2.74	0.7
**B-MZ (%B)**	8.48	0.77	4.27	0.46	7.15	0.72	1.24	0.12	0.89	0.1	1.52	0.13
**B-MZ (%LC)**	0.56	0.52	0.7	0.65	0.8	0.69	0.26	0.26	0.17	0.16	0.41	0.42
**B-SM (%B)**	17.2	0.74	2.47	0.06	2.09	0.05	2.29	0.06	2.32	0.06	3.7	0.07
**B-SM (%LC)**	1.13	0.51	0.4	0.12	0.23	0.06	0.48	0.17	0.44	0.14	0.99	0.44
**B-SM-IgA (%B)**	3.86	0.52	0.64	0.04	0.04	0.01	0.77	0.04	1.2	0.1	1.07	0.08
**B-SM-IgG (%B)**	9.09	0.73	1.78	0.11	1.78	0.11	1.19	0.07	0.82	0.06	2.33	0.17
**PBs (%B)**	3.96	0.88	0.34	0.06	0.09	0.03	1.03	0.35	1.08	0.38	4.22	0.89
**B-trans-b (%B)**	3.34	0.47	2.76	0.38	0.27	0	4.34	0.61	1.39	0.13	2.24	0.31
**NK (%LC)**	20.9	0.62	13.4	0.3	7.88	0.07	12.8	0.3	10.2	0.16	-	-
**NK- 1 (%LC)**	2.15	0.92	2.63	0.94	0.09	0	0.81	0.42	1.49	0.81	-	-
**NK-2 (%LC)**	16.6	0.72	8.92	0.4	4.95	0.13	7.56	0.33	6.47	0.22	-	-
**NK-3 (%LC)**	1.68	0.32	0.96	0.13	2.54	0.48	3.12	0.56	1.44	0.29	-	-
**Mono-class (%APC)**	21.3	0.07	65.6	0.39	85.8	0.97	17.8	0.06	17.4	0.06	-	-
**mDCs (%APC)**	40.6	0.93	-	-	7.83	0.12	35	0.92	13.9	0.41	-	-
**mDC-CD16+ (%APC)**	32.1	0.98	-	-	3.31	0.12	8.14	0.51	1.84	0.07	-	-
**mDC-CD16- (%APC)**	8.48	0.75	-	-	4.46	0.34	26.7	0.98	12.1	0.83	-	-
**pDCs (%APC)**	5.56	0.9	-	-	0.66	0.1	3.51	0.76	7.04	0.95	-	-
**LDNs (%APC)**	-	-	-	-	-	-	-	-	-	-	-	-
**Mono-non-class (%APC)**	9.65	0.96	1.29	0.11	0.34	0.05	0.74	0.08	0.47	0.07	-	-

CTLA4 Family

	Normal		Proband CTLA4+-		Brother CTLA4+-		Daughter (WT)	
	Raw	Cent.	Raw	Cent.	Raw	Cent.	Raw	Cent.
**CD3 (%LCs)**	81.9	0.95	57.5	0.2	82.4	0.95	81	0.94
**CD4 (%LCs)**	48.5	0.81	32.6	0.35	55.7	0.92	48	0.76
**CD4-Act (%CD4)**	0.71	0.33	5.65	1	1.55	0.81	0.3	0.04
**CD4-Naive (%CD4)**	62.7	0.78	38.5	0.32	8.7	0.02	85	1
**CD4-TCM (%CD4)**	34.6	0.46	52.1	0.86	34.7	0.46	13	0.05
**CD4-TEM (%CD4)**	2.55	0.02	8.39	0.44	47.9	0.98	2	0
**CD4-TEMRA (%CD4)**	0.15	0.05	0.96	0.51	8.65	0.88	0.3	0.1
**CD8 (%LC)**	29.5	0.86	20.7	0.47	20	0.44	22	0.58
**CD8-Act (%CD8)**	0.42	0.08	4.89	0.9	1.48	0.51	0.2	0
**CD8-Naive (%CD8)**	44.6	0.66	38.2	0.56	6.69	0.1	64	0.95
**CD8-TCM (%CD8)**	19.3	0.88	12.5	0.69	22.9	0.95	8.7	0.46
**CD8-TEM (%CD8)**	23.9	0.49	17.1	0.29	51.6	0.97	23	0.48
**CD8-TEMRA (%CD8)**	12.1	0.21	32.2	0.61	18.8	0.42	3.7	0.02
**CD4-Exh (%CD4)**	0.56	0.51	1.25	0.76	8.36	0.99	0.2	0.29
**X5-Th1 (%CD4)**	2.61	0.61	3.77	0.78	4.86	0.91	0.6	0.04
**X5-Th1/17 (%CD4)**	2.83	0.97	0.37	0.3	1.49	0.82	0.1	0.13
**X5-Th2 (%CD4)**	1.84	0.43	0.87	0.04	7.33	0.97	1.4	0.25
**X5-Th17 (%CD4)**	4	0.94	0.9	0.27	6.25	0.98	0.5	0.21
**X5-Th (%CD4)**	11.3	0.85	5.92	0.39	19.9	0.98	2.7	0.04
**Tfh-eff (%CD4)**	2.04	0.81	2.6	0.91	9.73	1	0.4	0.14
**Tfh-eff (%CXCR5)**	18.4	0.63	44	1	48.8	1	13	0.3
**Tfh-mem (%CD4)**	8.8	0.84	3.16	0.17	9.46	0.88	2.3	0.05
**Tfh-mem (%CXCR5)**	79.6	0.31	53.3	0	47.5	0	85	0.73
**Th1 (%CD4)**	6.64	0.26	25.7	0.91	30.7	0.97	3.3	0.04
**Th1-17 (%CD4)**	6.9	0.53	1.5	0.09	5.83	0.47	1.2	0.08
**Th2 (%CD4)**	6.78	0.4	13.7	0.78	8.92	0.6	5.9	0.27
**Th17 (%CD4)**	4.28	0.53	2.77	0.33	5.96	0.73	2.4	0.27
**Treg (%CD4)**	6.31	0.79	18.1	1	13.5	0.98	4.3	0.41
**CD19 (%LC)**	8.9	0.41	0.74	0.02	8.05	0.34	7.9	0.31
**B-CD21lo (%B)**	5.53	0.06	54	1	11.5	0.3	13	0.4
**B-mem (%B)**	12.4	0.21	16.8	0.41	19.7	0.51	11	0.14
**B-mem (%LC)**	1.1	0.16	0.12	0	1.59	0.34	0.9	0.13
**B-MZ (%B)**	7.17	0.73	3.47	0.38	8.89	0.8	5	0.5
**B-MZ (%LC)**	0.64	0.62	0.026	0.02	0.72	0.65	0.4	0.42
**B-SM (%B)**	3.65	0.07	0.99	0.03	9.03	0.44	4.7	0.15
**B-SM (%LC)**	0.32	0.1	0.0073	0	0.73	0.3	0.4	0.11
**B-SM-IgA (%B)**	0.9	0.05	0	0	2.6	0.28	1	0.05
**B-SM-IgG (%B)**	2.65	0.22	0	0	5.77	0.57	3.7	0.35
**PBs (%B)**	0.79	0.25	5.45	0.93	5.09	0.93	0.4	0.07
**B-trans-b (%B)**	2.4	0.35	1.49	0.14	1.24	0.12	1.1	0.09
**NK (%LC)**	7.319	0.05	25.63	0.8	7.81	0.07	6.3	0.04
**NK- 1 (%LC)**	0.31	0.05	3.48	0.97	0.32	0.05	0.3	0.03
**NK-2 (%LC)**	5.43	0.14	11.2	0.54	6.9	0.29	5	0.13
**NK-3 (%LC)**	1.49	0.29	8.67	0.83	0.4	0.02	0.3	0
**Mono-class (%APC)**	84	0.96	83.2	0.95	86.7	0.98	75	0.72
**mDCs (%APC)**	-	-	-	-	-	-	-	-
**mDC-CD16+ (%APC)**	-	-	-	-	-	-	-	-
**mDC-CD16- (%APC)**	-	-	-	-	-	-	-	-
**pDCs (%APC)**	-	-	-	-	-	-	-	-
**LDNs (%APC)**	0.0057	0.12	5.88	0.93	0.1	0.62	2.6	0.93
**Mono-non-class (%APC)**	1.49	0.13	2.37	0.29	0.75	0.08	2.1	0.22

CARD 11 Subjects

	Proband A CARD11		Husband A WT		Proband B CARD11		Husband B WT		Mother B WT		Father B WT	
	Raw	Cent.	Raw	Cent.	Raw	Cent.	Raw	Cent.	Raw	Cent.	Raw	Cent.
**CD3 (%LCs)**	94.1	1	86.5	1	86.4	1	64.5	0.4	64.1	0.36	69.2	0.55
**CD4 (%LCs)**	37.6	0.47	48.2	0.8	61	0.97	39.8	0.56	41.5	0.58	59.2	0.95
**CD4-Act (%CD4)**	1.78	0.87	1.29	0.74	0.21	0	0.24	0.02	0.58	0.26	0.17	0
**CD4-Naive (%CD4)**	66	0.85	58	0.68	74.3	0.97	63.4	0.79	17.3	0.06	83	1
**CD4-TCM (%CD4)**	29	0.27	24.8	0.2	21	0.11	29.8	0.3	71.7	1	16	0.06
**CD4-TEM (%CD4)**	4.06	0.06	11.3	0.62	3.65	0.03	6.14	0.25	10.5	0.56	0.87	0
**CD4-TEMRA (%CD4)**	0.86	0.46	5.91	0.84	1.04	0.52	0.65	0.37	0.5	0.25	0.12	0.03
**CD8 (%LC)**	48.5	1	31.8	0.91	21.8	0.54	19.7	0.43	18.5	0.4	8.24	0.05
**CD8-Act (%CD8)**	9.21	0.97	2.05	0.69	0.5	0.12	0.51	0.13	1.75	0.62	0.85	0.27
**CD8-Naive (%CD8)**	8.61	0.14	36.6	0.55	80.4	1	42.1	0.61	18.5	0.29	41.4	0.6
**CD8-TCM (%CD8)**	2.03	0.02	9.47	0.53	5.17	0.19	24.5	0.95	40.3	1	15.6	0.83
**CD8-TEM (%CD8)**	5.65	0.02	17.3	0.3	4.79	0.02	22.6	0.47	32.3	0.72	18.1	0.32
**CD8-TEMRA (%CD8)**	83.7	1	36.6	0.67	9.59	0.14	10.7	0.17	8.86	0.11	25	0.46
**CD4-Exh (%CD4)**	1.17	0.75	1.56	0.82	0.13	0.18	0.49	0.47	0.4	0.42	0.18	0.29
**X5-Th1 (%CD4)**	2.75	0.63	4.35	0.88	2.08	0.44	-	-	7.43	0.98	-	-
**X5-Th1/17 (%CD4)**	1.49	0.82	0.55	0.4	0.17	0.15	-	-	0.32	0.27	-	-
**X5-Th2 (%CD4)**	0.6	0	3.78	0.91	1.76	0.43	-	-	3.62	0.9	-	-
**X5-Th17 (%CD4)**	0.77	0.26	1.21	0.37	0.14	0.09	-	-	0.5	0.21	-	-
**X5-Th (%CD4)**	5.61	0.36	9.89	0.68	4.15	0.14	5.97	0.4	11.9	0.88	0.47	0.02
**Tfh-eff (%CD4)**	1.71	0.77	1.73	0.77	0.66	0.26	0.57	0.22	3.09	0.94	0.04	0
**Tfh-eff (%CXCR5)**	30.5	1	17.5	0.6	15.9	0.47	9.64	0.16	26	0.93	8.21	0.13
**Tfh-mem (%CD4)**	3.74	0.24	7.94	0.73	3.45	0.24	5.33	0.53	8.61	0.78	0.42	0.02
**Tfh-mem (%CXCR5)**	66.8	0.02	80.3	0.38	83.2	0.56	89.4	0.84	72.6	0.09	90.3	0.86
**Th1 (%CD4)**	9.11	0.43	9.65	0.48	9.75	0.5	-	-	37.7	0.98	-	-
**Th1-17 (%CD4)**	4.16	0.33	5	0.37	1.62	0.1	-	-	7.05	0.53	-	-
**Th2 (%CD4)**	8.13	0.59	4.38	0.13	6.19	0.31	-	-	17.1	0.9	-	-
**Th17 (%CD4)**	2.49	0.28	3.06	0.37	1.12	0.1	-	-	2.81	0.33	-	-
**Treg (%CD4)**	3.26	0.26	7.49	0.9	6.13	0.74	4.22	0.38	5.08	0.56	4.25	0.38
**CD19 (%LC)**	2.83	0.03	5.01	0.1	3.06	0.04	11.1	0.62	10.9	0.6	11.4	0.63
**B-CD21lo (%B)**	6.73	0.08	12.2	0.33	5.24	0.05	10.8	0.27	9.53	0.17	8.9	0.12
**B-mem (%B)**	6.31	0.04	19.6	0.5	10.9	0.15	17.1	0.41	25.5	0.66	10.8	0.14
**B-mem (%LC)**	0.18	0.01	0.98	0.14	0.33	0.02	1.9	0.47	2.78	0.71	1.23	0.23
**B-MZ (%B)**	1.99	0.18	6.3	0.6	0.63	0.08	2.23	0.2	3.73	0.4	2.44	0.21
**B-MZ (%LC)**	0.06	0.04	0.32	0.35	0.02	0.02	0.25	0.24	0.41	0.42	0.28	0.31
**B-SM (%B)**	3.84	0.08	13.1	0.64	7.43	0.32	12.4	0.62	17.2	0.74	5.17	0.17
**B-SM (%LC)**	0.11	0.02	0.66	0.28	0.23	0.06	1.37	0.6	1.88	0.79	0.59	0.24
**B-SM-IgA (%B)**	1.26	0.11	4.32	0.57	1.29	0.12	3.14	0.45	8.11	0.9	2.35	0.22
**B-SM-IgG (%B)**	0.64	0.05	3.86	0.35	4.24	0.37	8.47	0.72	8.25	0.7	2.31	0.17
**PBs (%B)**	4.43	0.89	9.35	0.95	5.87	0.93	2.57	0.78	3.36	0.88	2.31	0.74
**B-trans-b (%B)**	19.6	1	3.79	0.55	4.41	0.61	4.71	0.67	3.34	0.47	8.65	0.93
**NK (%LC)**	2.13	0	7.19	0.05	8.88	0.09	22.4	0.68	21.3	0.62	17.3	0.47
**NK- 1 (%LC)**	0.36	0.07	0.53	0.12	1.08	0.61	0.91	0.53	1.73	0.88	0.89	0.51
**NK-2 (%LC)**	0.24	0	3.19	0.07	6.14	0.19	19.2	0.78	14.6	0.69	12.1	0.56
**NK-3 (%LC)**	1.44	0.29	3.22	0.57	0.75	0.09	1.68	0.32	2.11	0.42	1.5	0.29
**Mono-class (%APC)**	69.7	0.55	74.5	0.72	61.5	0.32	49.3	0.21	72.4	0.65	78.7	0.88
**mDCs (%APC)**	10.8	0.26	14.7	0.5	14.8	0.53	30	0.91	11.7	0.28	9.42	0.17
**mDC-CD16+ (%APC)**	4.27	0.15	11.6	0.69	9.26	0.56	19.6	0.93	5.75	0.24	5.61	0.23
**mDC-CD16- (%APC)**	6.52	0.61	3.02	0.17	5.46	0.53	10.2	0.82	5.93	0.56	3.79	0.28
**pDCs (%APC)**	1.01	0.2	0.84	0.14	5.33	0.9	8.44	0.97	4	0.84	0.87	0.14
**LDNs (%APC)**	0.42	0.8	0.0075	0.14	0.1	0.62	0.28	0.74	0	0.01	0	0.01
**Mono-non-class (%APC)**	11.3	0.97	5.95	0.82	4.47	0.67	3.46	0.44	3.92	0.5	7.06	0.9
